# Endocan, sepsis, pneumonia, and acute respiratory distress syndrome

**DOI:** 10.1186/s13054-018-2222-7

**Published:** 2018-10-26

**Authors:** Nathalie De Freitas Caires, Alexandre Gaudet, Lucie Portier, Anne Tsicopoulos, Daniel Mathieu, Philippe Lassalle

**Affiliations:** 1 University of Lille, U1019—UMR 8204—Center for Infection and Immunity of Lille, F-59000 Lille, France; 2 CNRS, UMR 8204, F-59000 Lille, France; 3 INSERM, U1019, F-59000 Lille, France; 4 Institut Pasteur de Lille, F-59000 Lille, France; 5 Lunginnov, 1 rue du Pr Calmette, F-59000 Lille, France; 6 CHU Lille, Pôle de Réanimation, Hôpital Roger Salengro, F-59000 Lille, France; 7 CHRU de Lille, Clinique des maladies respiratoires, Hôpital Calmette, F-59000 Lille, France

**Keywords:** Endocan, Sepsis, Acute respiratory distress syndrome, Pneumonia

## Abstract

Acute respiratory distress syndrome (ARDS) and hospital-acquired pneumonia (HAP) are major problems of public health in intensive care units (ICUs), occurring in 15% of critically ill patients. Among the factors explaining ARDS development, sepsis is known as a frequent cause. Sepsis, ARDS, and HAP increase morbidity, mortality, length of stay in the ICU, and the overall costs of healthcare. The major challenge remains to identify accurately among critically ill patients those at risk of poor outcomes who could benefit from novel therapies. Endocan is released by the pulmonary endothelium in response to local or systemic injury. It inhibits mainly leukocyte diapedesis rather than leukocyte rolling or adhesion to the endothelial cells both in vitro and in vivo. Endocan was evaluated in 25 clinical reports, including 2454 critically ill patients and 452 healthy controls. The diagnostic value of endocan for sepsis or sepsis severity was equal to procalcitonin but its prognostic value was better. A predictive value for postoperative pneumonia was evidenced in two studies, and a predictive value for ARDS in four studies from three independent centers. This review presents an overview of the structure, expression, and functions of endocan. We also hereby summarize the potential applications of endocan in the prediction and prognosis of ARDS and HAP, as well as in the prognosis of sepsis.

## Background

Hospital-acquired pneumonia (HAP) and acute respiratory distress syndrome (ARDS) are major lung injuries that commonly occur in intensive care unit (ICU) patients. In ICU patients, HAP is mainly represented by postoperative pneumonia (POP) and ventilator-associated pneumonia (VAP), respectively defined as pneumonia occurring at least 48 h after surgery or tracheal intubation. Although inconsistent, the incidence of HAP may reach up to 25% in patients undergoing cardiac surgery, thus leading to increased morbidity and hospital length of stay [1]. The delay observed between surgery and the clinical onset of pneumonia, leading to late initiation of antibiotics, might represent one possible explanation for this increased morbidity. Therefore, early identification of patients who are the most likely to develop HAP could allow initiation of preemptive treatments prior to the clinical onset of pneumonia.

ARDS is another frequent condition among ICU patients. It results from an inflammatory state that affects fluid leakage and leukocyte recruitment into the air spaces, thus leading to major hypoxemia. Although many results from clinical trials have led to improved treatment of ARDS, this condition is still responsible for high levels of mortality. There has for several years been a shift of paradigm about the management of ARDS, considering that preemptive therapeutic approaches, targeting lung inflammation before its clinical onset, could lead to major improvement of its prognosis [2]. Thus, a better understanding of molecular regulatory pathways leading to ARDS could be of high interest, especially in acute systemic inflammatory states such as sepsis, which has been identified as a risk factor for ARDS [3, 4].

Sepsis is a life-threatening condition commonly observed in critically ill patients, associated with high mortality rates reaching levels above 25% in septic shock mainly resulting from the development of multiorgan failure. However, sepsis is a heterogeneous condition, encompassing at the same time patients with various prognoses, with either favorable evolutions or poor outcomes [5, 6]. The detection of subjects who exhibit the highest risk of poor outcomes among septic patients might help to improve their prognosis through early adaptation of their management.

Endocan, or endothelial cell specific molecule-1, is a 50-kDa proteoglycan mainly expressed by lung endothelial cells, whose secretion in the bloodstream is upregulated by proinflammatory cytokines (IL-1β, TNF-α) and bacterial LPS [7]. The biological role of endocan results from its ability to link with the integrin LFA-1, leading to the inhibition of its interaction with its endothelial ligand ICAM-1 [8–10]. This phenomenon leads to an inhibition of leukocyte diapedesis, explaining a potential anti-inflammatory effect of endocan during acute lung injury. Several convergent reports confer to indicate blood endocan as a predictive marker for HAP and ARDS development.

In this review, we present a summary of the structure, expression, and functions of endocan. We also report its potential applications in the prediction and prognosis of ARDS and HAP, as well as in the prognosis of sepsis.

## Main text

### Structure of the *esm1* gene

The human endocan cDNA was first cloned in our laboratory in 1996 from a HUVEC cDNA library (Genbank accession number X89426). Cloning of its gene was achieved in 1999 (Genbank accession numbers AJ401091 and AJ401092). The mouse endocan cDNA and gene were also cloned first in our laboratory in 1999 and 2000 (Genbank accession numbers AJ249354 and AJ416379).

Initially called *esm1* for endothelial cell specific molecule-1, the molecule was renamed endocan in 2001 based on its specific endothelial expression and its proteoglycan nature. Thus, the gene name remains *esm1*, and its product name is endocan.

**Fig. 1:**
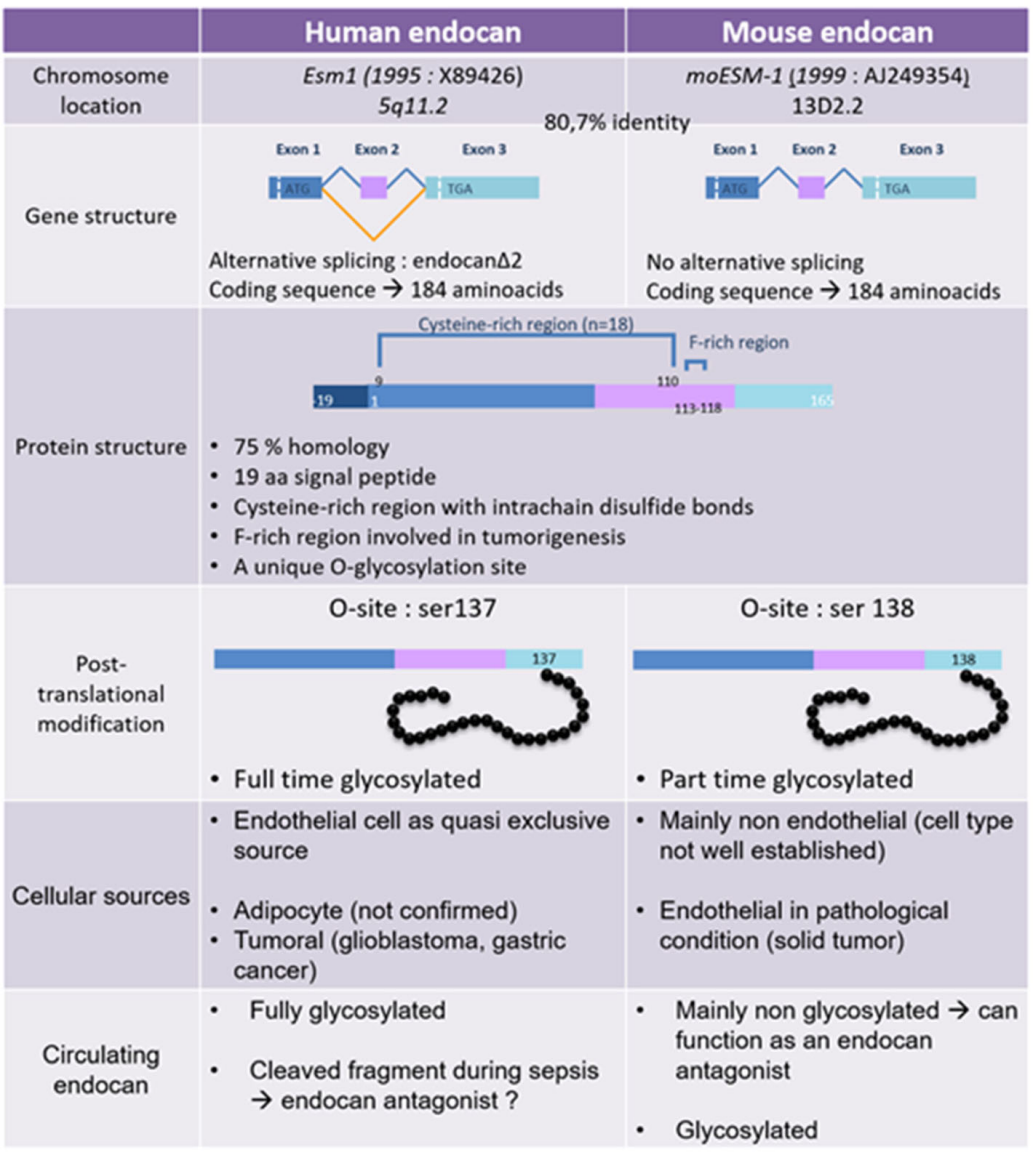
Comparative structure and expression of human and mouse endocan

The human *esm1* gene is located on chromosome 5 at position q11.2. It covers 12 kilobases and is organized into three exons (Fig. 1). The *esm1* genes from other species span equivalent sizes and possess the same number of exons [7].

The mRNA from all species encodes for a propeptide including a signal peptide of 19 amino acids and a mature polypeptide of typically 165 amino acids in the vast majority of mammalians.

### Homologies

No clear structural evidence allows one to include endocan in the existing family of proteoglycans: no lectin site is present, and there is no central domain containing leucine-rich repeats, a characteristic of the small leucine-rich proteoglycans [11]. However, endocan is rich in cysteines with 10.9% of the protein encompassing cysteine residues [6]. Interestingly, all 18 cysteines are concentrated in the 110-amino-acid N-terminal region of the protein (Fig. 1). Endocan shows a 15–28% identity with members of the insulin-like growth factor (IGF) binding-protein superfamily, characterized by their cysteine-rich content and their affinity for IGF [7, 12]. However, no evidence for the binding of IGF by endocan has yet been demonstrated [8]. The best protein similarities are found with the human β_2_ integrins (cysteine-rich tandem repeats: 27.6% identity and 54.6% similarity) [7].

### Polymorphism

Based on the search for variants of the human *esm1* gene on the ExAC site, the risk of finding a homozygous variant is estimated among the European population to be less than 2 individuals per million, as summarized in Table 1. Note that this risk is equivalent in the Asian population. On the other hand, the risk is twice as high in the African population and five times higher in the Latino population.

**Table 1:** Polymorphism of endocan

	Population			
	Caucasian	Latino	Asian	African
Allele number sequenced	66,656	11,558	25,162	10,406
Variant number	86	34	40	20
Variant frequency	0.00129	0.002942	0.00159	0.001922
Homozygous frequency	1/600,733	1/115,560	1/395,704	1/270,712
Ratio	1.00	5.20	1.52	2.22

### Structure of endocan

The human endocan is a soluble proteoglycan consisting of a mature protein of 165 amino acids ​​and a chondroitin/dermatan sulfate glycan chain of 15–40 kDa covalently linked to the protein on serine 137 [7, 13, 14]. Circulating human endocan is fully glycosylated.

The protein core contains several domains respectively encoded by exons 1, 2, and 3, as shown in Fig. 1 [13, 15].

The mouse endocan encodes for a mature polypeptide of 165 amino acids with 74% homology with its human counterpart and all conserved structural domains (Fig. 1) [16]. But recombinant and endogenous circulating mouse endocan are both much less glycosylated than human endocan.

### Expression, distribution, and catabolism of endocan

Human endocan is produced specifically by the vascular endothelium and preferentially by the pulmonary and kidney endothelial cells [7]. Tumoral cells can sometimes express endocan (grade III–IV glioblastoma or pituitary adenomas) [17, 18]. Endocan does not belong to the endothelial glycocalix as other proteoglycans like syndecans and circulates freely at about 1 ng/ml in the bloodstream [19, 20].

Mouse endocan was found expressed in endothelial tip cells in several retinal angiogenesis models [21], and in tumor endothelial cells from xenogenic tumors [22]. However, mouse endocan also appears to be produced by some nonendothelial cells, not yet defined, from the lung, kidney, and spleen [22]. Mouse endocan also circulates freely at a baseline concentration of 1 ng/ml [16].

The catabolism of endocan is not well established. One catabolic pathway involves a proteolytic degradation by neutrophil cathepsin G, especially generating one major catabolite of 14 kDa detected in serum from healthy subjects and septic patients [23]. Endocan might also be eliminated through hepatic metabolism, as the increase of blood endocan inversely correlates with the degree of liver deficiency [24].

### Endocan gene regulation

Endocan is continually synthetized and secreted under physiological conditions. However, its synthesis and secretion are both upregulated upon endothelial cell stimulation by TNF-α, VEGF, and glycated serum albumin [25, 26]. These increases are observed in vitro 1 h after stimulation for endocan mRNA and 4 h after stimulation for endocan secretion. Then, endocan mRNA and secretion remain high for 24 h, before progressively decreasing to baseline levels [26]. In contrast, basal and TNF-α-induced endocan synthesis and secretion are inhibited by activators of JAK2/STAT3-snail pathways, such as IFN-γ [26], or by activators of PI3 kinase/Akt/FOXO1-ZEB pathways such as PDGF, angiotensin II, endothelin 1, insulin, or hyperglycemia [27, 28].

### Inflammation

Upregulation of endocan by proinflammatory cytokines led to suspecting a role in regulating the inflammatory reaction. Indeed, it was initially found that endocan binds to LFA-1 with high affinity (kD_A_ = 18 nM) and inhibits its interaction with the adhesion molecule ICAM-1 [8]. This was later confirmed by intravital microscopy and biomimetic microfluidic assay showing that endocan alleviates leukocyte rolling and transmigration, but not firm adhesion [10].

In animal models, calpain inhibitor pretreatment of endotoxemic rats reduces the severity of endoxemia at 6 h, and is associated with an increase of blood endocan levels and a reduction in leukocyte rolling [29]. In a murine endotoxin-induced acute lung injury model, pretreatment with endocan decreases TNF-α, IFN-γ, IL-1β, and IL-6 levels and alleviates pulmonary epithelial cell apoptosis [30]. Blockade of endocan in mice bearing tumor xenografts induces a stromal inflammation and slows tumor growth [9]. An *esm1* gene deficiency impairs leukocyte extravasation at the transmigration step. In a peritonitis assay, *esm1*^*−/−*^ mice exhibited a significant decrease in leukocyte extravasation when compared with control littermates [31]. By intravital microscopy, the decreased leukocyte extravasation observed in *esm1*^*−/−*^ mice was solely attributable to reduced transmigration but not to adhesion or rolling [10].

This apparent discrepancy between functions of human and mouse endocan may be explained by the differences between their respective cellular sources and glycanation levels [32]. Indeed, by contrast with its human counterpart, mouse endocan is spontaneously produced by nonendothelial cells in the lung, kidney, and spleen. In healthy mice, the main circulating form of endocan is mainly nonglycosylated and nonendothelial, which is quite different from the main human circulating form, fully coming from endothelial cells and fully glycosylated. Each of these forms is suspected to possess opposite biological properties depending on the glycanic status: anti-inflammatory for the glycosylated form, proinflammatory for the nonglycosylated form.

Mouse endocan sounds more complex than its human counterpart. The glycanic status and the function of the true endothelial cell-derived mouse endocan is presently unknown.

### Clinical implications

The lung is sensitive to various infectious, toxic, pharmacologic, or immunologic agents. In response, the lung develops an inflammatory reaction which can compromise gas exchange and the patient’s outcome. During the course of inflammation, the pulmonary endothelial cells are solicited to participate in its regulation. In turn, blood endocan increases mainly through de-novo synthesis and secretion [26]. This increase of blood endocan occurs early and independently of any alveolocapillary lesion.

#### Endocan and community-acquired pneumonia

Very little is known about the expression of endocan during the course of pneumonia. A first study showed increased plasma endocan levels in 82 community-acquired pneumonia patients, which moderately correlate with clinical severity scores such as the Pneumonia Severity Index (*r* = 0.554, *p* < 0.001), CURB-65 (*r* = 0.510, *p* < 0.001), and Acute Physiology and Chronic Health Evaluation II (*r* = 0.447, *p* < 0.001), but not with CRP, white blood cells, or neutrophils [33]. The kinetics of endocan during the first 24 h after the diagnosis of pneumonia exhibited no change for the first 3 h, but decreased 24 h later [34]. No correlations were found between endocan and IL-6, IL-10, NGAL, Resistin, MPO, ICAM-1, and VCAM-1 either at 0 h or 24 h, except for VCAM-1 at 24 h (*ρ* = 0.52, *p* = 0.009) [34].

#### Prediction and prognosis of hospital-acquired pneumonia

Postoperative pneumonia (POP) is a hospital-acquired pneumonia that develops at least 48 h after surgery. The incidence of POP varies depending on the patient’s comorbidities and the type of surgery, but also on the diagnostic criteria used. It ranges from 2 to 25% of operated patients in cardiac surgery [1]. In addition, POP extends the average hospital length of stay from 4 to 9 days [1]. The diagnosis of POP remains difficult, based essentially on a bundle of clinical, radiological, and biological arguments. In a study enrolling patients undergoing cardiac surgery, C-reactive protein (CRP) values measured in patients evolving to VAP did not differ before day 3 following surgery from levels observed in subjects in whom VAP did not occur [35]. In another study, procalcitonin (PCT) did not allow accurate discrimination of POP until at least 3 days after cardiac surgery [36].

A total of 330 patients undergoing cardiac surgery with and without the use of cardiopulmonary bypass (CPB) have been investigated for blood endocan. All showed increased endocan levels peaking at 6–24 h post surgery, which then slowly declined yet were not returned to baseline concentrations at day 5 [37–40]. The endocan level in cardiac surgery with CPB peaked earlier at 6 h and was higher than that in patients undergoing off-pump surgery [40, 41]. No correlation was found between endocan levels at 6 h post surgery and the duration of CPB [40]. Higher perioperative concentrations of endocan were observed in patients with the longest duration of norepinephrine support (*p* = 0.007) [38]. Patients developing POP exhibited higher blood endocan levels before surgery and at 6 h post surgery than those who did not develop POP (Table 2) [42, 43]. Thus, endocan allowed earlier identification of patients evolving to POP than PCT or CRP [35, 36].

**Table 2:** Predictive value of endocan for postoperative pneumonia quantified with EndoMark H1

	Diagnostic value 6 h post surgery							
	POP/*n*	Cutoff point (ng/ml)	Se	Sp	PPV	NPV	AUC	*p*
Pilot study	5/20	15.90	0.80	1.00	1.00	0.76	0.840	< 0.01
Validation study	17/155	15.20	0.41	0.95	0.47	0.94	0.745	< 0.001

*AUC* area under the curve, *NPV* negative predictive value, *POP* postoperative pneumonia, *PPV* positive predictive value, *Se* sensitivity, *Sp* specificity

Blood endocan levels at 12 h, 24 h, or 48 h post surgery were less discriminant. In routine practice, the average time to a formal diagnosis of pneumonia after cardiac surgery is 4.6 ± 3.7 days [43]. Blood endocan > 15 ng/ml 6 h after cardiac surgery had a sensitivity of 80% and a specificity of 100% to predict POP [42].

Ventilator-associated pneumonia (VAP) is another hospital-acquired pneumonia that develops 48 h or longer after mechanical ventilation. The incidence of VAP increases with the duration of mechanical ventilation. VAP causes an increase of the mortality rate estimated at around 13% [44]. Endocan has been reported as a potential predictor of poor outcomes in VAP. Indeed, in a study enrolling 42 patients with VAP, blood endocan measured at day 1 and day 7 after the diagnosis of VAP was observed at higher levels in nonsurvivors than in survivors (day 1, 12.50 ± 2.74 ng/ml vs 10.35 ± 1.86 ng/ml, *p* < 0.05; day 7, 24.33 ± 7.14 ng/ml vs 5.93 ± 1.56 ng/ml, *p* < 0.001) [45].

#### Diagnosis and prognosis of sepsis

Sepsis remains a devastating problem in critically ill patients despite modern treatments, with an estimated mortality rate greater than 25% in patients suffering from septic shock [5, 6]. Early recognition and treatment might prevent the development of multiorgan failure, septic shock, and sepsis-related death [6]. Currently, only a combined evaluation of clinical signs as well as hemodynamic and laboratory parameters appears to improve objective and correct diagnostic decision-making for severe sepsis and septic shock [46].

Endothelial cells play a key role in the pathogenesis of sepsis by producing cytokines and chemotactic agents, and by expressing surface adhesion molecules, which induce migration of circulating leukocytes into tissues. Indeed, both endocan and angiopoietin-2 are endothelial-specific biomarkers which increase during sepsis, worsening into multiple organ dysfunction syndrome (MODS), and decrease when sepsis improves [47]. Endocan showed correlation with VCAM-1 and E-selectin but not with ICAM-1 [48]. Severe sepsis with endocan remaining > 6.28 ng/ml at days 1, 4, and 7 is associated with poor prognosis. Indeed, for each 1 ng/ml elevation of endocan levels, the fatality rate increased by 11.1% [48].

PCT represents the major biomarker used routinely for diagnosis/prognosis/monitoring of sepsis. The diagnosis/prognosis values of endocan have been compared to those of PCT in a total of 555 patients (Table 3). Accordingly, endocan appeared as a consistent good diagnostic criterion as well as PCT. However, the correlation between endocan and PCT remained poor. Indeed, no significant correlation was found between endocan and PCT in patients with bacteriemia [49, 50]. In severe sepsis, Scherpereel et al. [19] found no significant correlation between endocan and PCT, while Pauly et al. [51] reported a significant yet poor correlation between endocan and PCT (*r* = 0.17, *p* = 0.04).

**Table 3:** Diagnostic/prognostic values of endocan in sepsis

Diagnostic value	*n*		Cutoff point (ng/ml)	AUC	*p*	Corr	Reference
Sepsis	78	Endocan	1.26	0.89	< 0.01		[52]
		PCT	0.75	0.84	< 0.01	Yes	
28-day mortality		Endocan	4.37	0.91	< 0.01		
		PCT	7.68	0.76	< 0.01	Yes	
Bacteriemia	78	Endocan	1.70	0.66	0.02		[49]
		PCT	0.12	0.56	ns	No	
Bacteriemia	126	Endocan	2.05	0.83	< 0.001		[50]
		PCT	0.20	0.73	< 0.001	No	
Septic shock	150	Endocan	2.90	0.74	0.001		[51]
		PCT		0.83	0.001	Yes	
28-day mortality		Endocan		0.63	0.005		
		PCT		0.58	ns		
6-month mortality		Endocan		0.65	0.002		
		PCT		0.59	ns		
Organ failure on admission	60	Endocan		0.81	< 0.05		[53]
		PCT		0.79	< 0.05	ND	
MODS development in 48 h		Endocan		0.67	< 0.05		
		PCT		0.75	< 0.05	ND	
ICU mortality		Endocan		0.71	< 0.05		
		PCT		0.66	ns		
Septic shock	63	Endocan	3.00	0.78	< 0.02		[19]
		PCT		0.79	< 0.02	No	
30-day mortality		Endocan	5.50	0.81	< 0.02		
		PCT			ns		

*AUC* area under the curve, *Corr* existing correlation, *ICU* Intensive Care Unit, *MODS* multiorgan dysfunction syndrome, *ND* not determined, *ns* not significant, *PCT* procalcitonin

Furthermore, endocan appeared more accurate than PCT in the prognosis of sepsis. In two independent studies, ROC analysis showed that the area under the curve (AUC) of endocan to predict mortality at day 28 was observed at 0.91 by Zhao and Dong (vs 0.76 for PCT) [52] and at 0.63 by Pauly et al. (vs 0.58 for PCT) [51]. Moreover, the AUC for mortality at 6 months was found to be 0.65 for endocan and 0.59 for PCT [51]. Finally, the endocan and PCT AUC to predict mortality at ICU discharge was respectively observed as 0.71 and 0.66 [53].

#### Prediction and prognosis of ARDS

ARDS is a complex condition resulting from many dysregulated biological pathways. Several subtypes can be identified that respond differently to treatments according to the predominant etiology and mechanism of lung inflammation [54]. For example, blood IL-8 and TNF-α have been suggested for identifying a subtype of ARDS with exacerbated vascular permeability which can benefit from conservative fluid management [55].

ARDS should be considered as a heterogeneous syndrome where the potential interest of biomarkers to initiate targeted treatments is emerging. As a marker of lung endothelial response, endocan is expected to exert a protective role against acute lung inflammation. A total of 446 patients with or without ARDS at ICU admission have been tested currently for endocan. In polytrauma, severe sepsis, or septic shock patients without ARDS at admission, high levels of endocan were found predominantly in patients who did not develop ARDS [47, 56–58]. Consistently, blood endocan levels below 5.5 ng/ml in these critically ill patients without ARDS at admission were associated with a high risk of ARDS occurrence within 3 days (Table 4). In septic patients, endocan exhibited higher values than the Lung Injury Prediction Score (LIPS) to predict ARDS, with the AUC respectively calculated at 0.93 (95% CI 0.87–1.00; *p* < 0.001) for endocan and 0.55 (95% CI 0.39–0.71; *p* = 0.59) for the LIPS [58]. Conversely, high endocan levels in severe sepsis were associated with the need for mechanical ventilation independently of ARDS. However, this result might reflect a more important overall severity of sepsis rather than the development of lung inflammation [59].

**Table 4:** Predictive/prognostic value of endocan for ARDS

Predictive value	*n*	Clinical context	Cutoff point (ng/ml)	AUC	Se	Sp	*p*	Reference
ARDS onset	48	Polytrauma	< 5.00				0.03	[56]
ARDS onset	175	Sepsis	< 2.50				0.008	[47]
ARDS onset	19	Sepsis	< 3.55	0.92	0.85	1.00	< 0.05	[57]
ARDS onset	72	Sepsis	< 5.49	0.93	1.00	0.77	< 0.001	[58]
28-day mortality	42	ARDS	> 4.96	0.72	0.55	0.86	0.017	[60]
Improving	54	ARDS	< 6.00	0.66	0.85	0.41	< 0.01	[61]
Worsening	42		> 14.0		0.39	0.82		

*ARDS* acute respiratory distress syndrome, *AUC* area under the curve, *Se* sensitivity, *Sp* specificity

In patients with ARDS at admission, endocan was significantly higher in nonsurvivors than in survivors (median (IQR) 5.01 (2.98–8.44) ng/ml vs 3.01 (2.36–4.36) ng/ml, *p* = 0.017). Endocan could predict mortality of ARDS independently with a hazard ratio of 1.374 (95% CI 1.150–1.641) and an AUC of 0.715 (*p* = 0.017) by ROC analysis [60]. In another series of 90 patients with ARDS at admission, endocan concentrations on the day after ARDS diagnosis were significantly higher in patients with poor evolution than in those with good evolution, with the median (IQR) respectively observed as 12.0 (6.8–18.6) vs 7.2 (5.4–12.5) (*p* < 0.01) [61]. Consistently, high endocan levels (> 13 ng/ml) in mechanically ventilated ARDS correlated with failure to withdraw from mechanical ventilation [62].

**Fig. 2:**
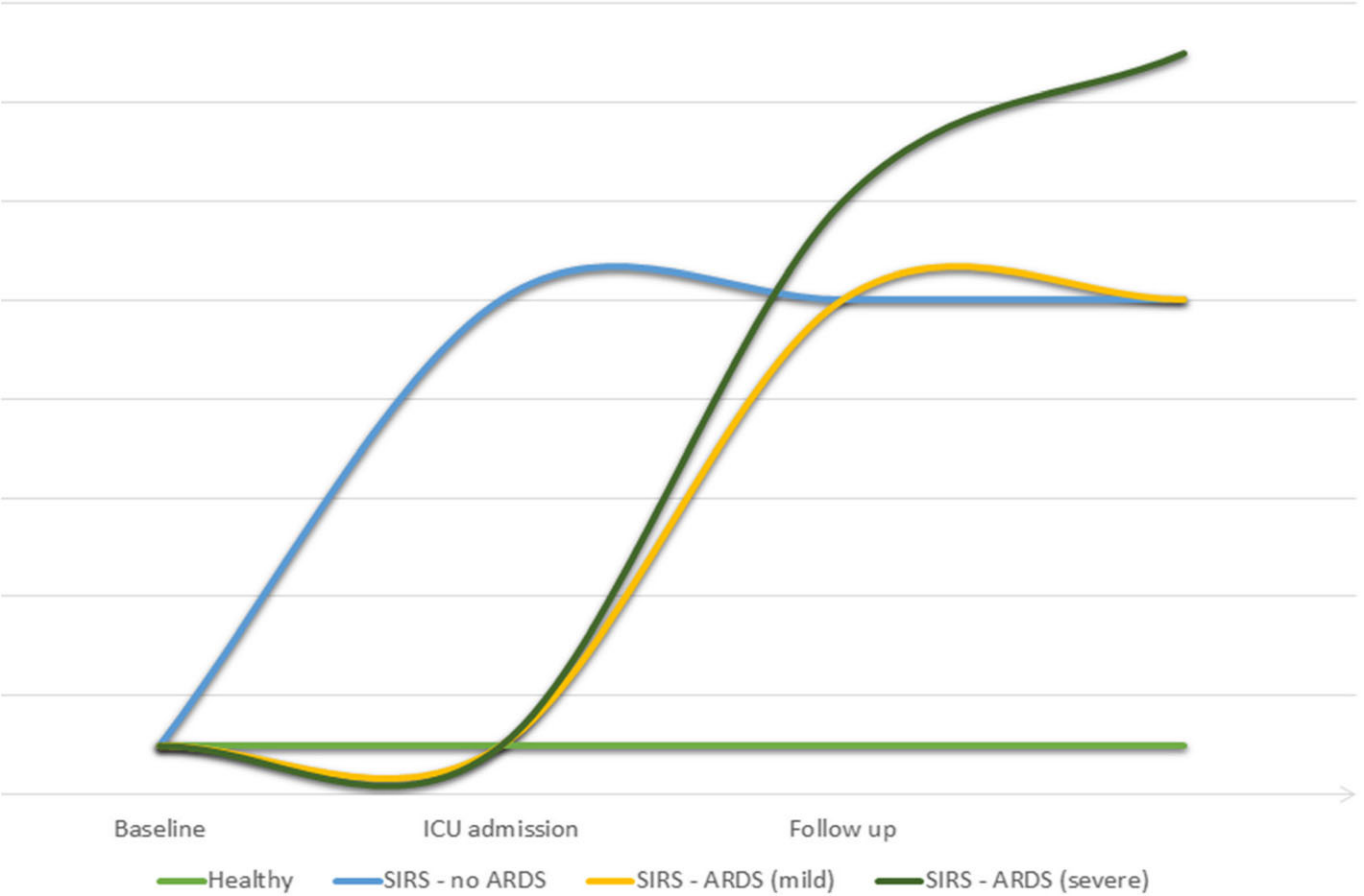
Hypothetical model of endocan kinetics in acute systemic and lung inflammatory states. ARDS acute respiratory distress syndrome, ICU intensive care unit, SIRS systemic inflammatory response syndrome

The aforestated observations suggest that endocan’s kinetics may be summarized as follows in the natural history of ARDS: endocan correlates with the overall severity of acute systemic inflammation; insufficient endocan levels observed in such cases prior to the development of lung injury seem to predict a higher risk of respiratory failure, consecutively to an insufficient anti-inflammatory effect; and increased endocan levels in constituted lung inflammation, as observed in ARDS, seem to correlate with poor outcomes. Altogether, these data suggest a delayed increase rather than an absence of an increase of blood endocan in patients who finally develop ARDS. Furthermore, this increase seems all the more marked when the ARDS is severe (Fig. 2).

#### Other critically ill patients

In critically ill hematology patients, endocan levels higher than 4.46 ng/ml at admission were associated with a higher cumulative probability of renal replacement therapy requirement (*p* = 0.006), a higher requirement of mechanical ventilation (*p* = 0.01), and a higher requirement of vasopressors throughout the ICU stay (*p* < 0.0001) [63]. Total endocan levels at admission were also independently associated with ICU mortality (odds ratio 1.39, 95% CI 1.06–1.83, *p* = 0.018).

## Conclusions

A better description of the phenotypical characteristics of ICU patients should help to differentiate patients with high risk of poor evolution from those who are likely to evolve favorably. Several authors underline the importance to separate these different phenotypes of patients, as they may respond differently to the same treatment: because they exhibit a phenotype corresponding to a particular physiopathological mechanism [55]; or because patients with the worst outcomes are the most likely to take the maximal benefit from a given treatment [64].

This approach may help to understand why most novel therapies that have been tested in the treatment of sepsis, HAP, or ARDS over the past decade have failed to show any benefit for ICU patients.

In sepsis, the association between the development of ARDS and paradoxically low endocan levels prior to its onset might be useful to identify patients exhibiting excessive leukocyte recruitment. This could correspond to a particular phenotype that could take maximal benefit from therapies modulating unbalanced inflammation, such as corticosteroids. Conversely, excessive endocan levels in patients with constituted ARDS could also be the witness of unbalanced pulmonary inflammation, thus indicating particular treatments such as corticosteroids or restrictive vascular filling.

In HAP, increased levels of endocan, combined with other parameters, could allow one to target patients potentially eligible for early antimicrobial therapy, started before the onset of pneumonia.

Of course, it is currently unknown whether abnormal endocan levels will eventually help to guide the early initiation of personalized treatments in ICU patients. However, current data give a rationale to explore these hypotheses.

## Abbreviations

ARDS: Acute respiratory distress syndrome
AUC: Area under the curve
CPB: Cardiopulmonary bypass
CRP: C-reactive protein
HAP: Hospital-acquired pneumonia
HUVEC: Human umbilical vein endothelial cell
ICU: Intensive care unit
IGF: Insulin-like growth factor
LIPS: Lung Injury Prediction Score
MODS: Multiple organ dysfunction syndrome
PCT: Procalcitonin
POP: Postoperative pneumonia
VAP: Ventilator-associated pneumonia
